# Effects of plasma on polyethylene ﬁber surface for prosthodontic application

**DOI:** 10.1590/1678-775720150260

**Published:** 2015

**Authors:** Silvana Marques Miranda SPYRIDES, Maíra do PRADO, Joyce Rodrigues de ARAUJO, Renata Antoun SIMÃO, Fernando Luis BASTIAN

**Affiliations:** 1 - Universidade Federal do Rio de Janeiro, Faculdade de Odontologia, Departamento de Prótese e Materiais Dentários; Departamento de Engenharia Metalúrgica e de Materiais, Rio de Janeiro, RJ, Brasil.; 2 - Universidade Federal do Rio de Janeiro, Departamento de Engenharia Metalúrgica e de Materiais, Rio de Janeiro, RJ, Brasil.; 3 - Instituto Nacional de Metrologia, Qualidade e Tecnologia, Laboratório de Fenômenos de Superfície, Duque de Caxias, RJ, Brasil.

**Keywords:** Chemical analysis, Plasma gases, Dental prosthesis, Topography

## Abstract

Plasma technology has the potential to improve the adherence of fibers to polymeric matrices, and there are prospects for its application in dentistry to reinforce the dental particulate composite.

**Objectives:**

This study aimed to investigate the effect of oxygen or argon plasma treatment on polyethylene ﬁbers.

**Material and Methods:**

Connect, Construct, InFibra, and InFibra treated with oxygen or argon plasma were topographically evaluated by scanning electron microscopy (SEM), and chemically by X-ray photoelectron spectroscopy (XPS). For bending analysis, one indirect composite (Signum) was reinforced with polyethylene fiber (Connect, Construct, or InFibra). The InFibra fiber was subjected to three different treatments: (1) single application of silane, (2) oxygen or argon plasma for 1 or 3 min, (3) oxygen or argon plasma and subsequent application of silane. The samples (25x2x2 mm), 6 unreinforced and 60 reinforced with fibers, were subjected to three-point loading tests to obtain their flexural strength and deflection. The results were statistically analyzed with ANOVA and the Bonferroni correction for multiple comparison tests.

**Results:**

SEM analysis showed that oxygen and argon plasma treatments promote roughness on the polyethylene fiber surface. X-ray photoelectron spectroscopy (XPS) analysis shows that both plasmas were effective in incorporating oxygenated functional groups. Argon or oxygen plasma treatment affected the flexural strength and deflection of a fiber reinforced composite. The application of silane does not promote an increase in the flexural strength of the reinforced composites.

**Conclusions:**

Oxygen and argon plasma treatments were effective in incorporating oxygenated functional groups and surface roughness. The highest strength values were obtained in the group reinforced with polyethylene fibers treated with oxygen plasma for 3 min.

## INTRODUCTION

Fiber-reinforced composites, initially used in fixed partial dentures and periodontal splinting, have been widely used in dentistry. Currently, they are also utilized in complete and partial crowns, root posts, and orthodontic appliances^10^ ^,^ ^23^. The mechanical and physical properties of fiber-reinforced composites largely depend on the properties and structure of the matrix/fiber interface. The difference between the elastic properties of the matrix and the fibers influences the load transfer through the interface^8^. Fiber-reinforced composites exhibit particular mechanical properties, different from those of neat fibers and non-reinforced particulate composites^7^ ^,^ ^9^.

Polyethylene fiber has specific strength and fracture toughness^29^. It also has a high elastic modulus, high tensile strength, low density, good biocompatibility^15^ ^,^ ^16^ ^,^ ^19^, high impact resistance, and flexibility^12^. On the other hand, it has some negative characteristics, such as a smooth and hydrophobic surface, a low surface energy, and a low strength under compression, and is chemically inert with non-polar surfaces, causing an inadequate interfacial adherence of the fiber with several polymeric matrices^12^ ^,^ ^16^ ^,^ ^19^ ^,^ ^28^. A good adhesion of the fiber-matrix interface is the basic principle for stress distribution in a composite, promoting strength and toughness^15^ ^,^ ^18^. Spyrides and Bastian^26^ (2004) showed that there were no statistically significant differences between the flexural strength and elastic modulus of composites reinforced with fiberglass or polyethylene fibers. The authors also observed that the fracture toughness of the polyethylene fiber-reinforced composites was superior to the fiberglass reinforced composite. In the same study, the polyethylene fiber-reinforced composite showed elasto-plastic behavior, and the fracture observed was by delamination, indicating that this material can be improved through interface studies.

Silanization is known to promote the inclusion of functional groups on polyethylene which can promote its adhesion to different matrices^2^ ^,^ ^25^. The treatment of polyethylene fibers with gaseous plasma is a process that can also improve their adhesion to polymeric matrices in composites by introducing polar groups containing oxygen or increasing surface roughness^12^ ^,^ ^16^ ^,^ ^17^ ^,^ ^24^.

Plasma technology has potential to improve the adherence of fibers to polymeric matrices, and the possibility of its application in dentistry to reinforce the dental particulate composites encouraged the present study. The aim of this study was to evaluate the effect of induced roughness, as well as the introduction of different chemical species to the polyethylene fiber, to improve the mechanical properties of reinforced composites. This was achieved by treating the polyethylene fibers with oxygen or argon cold plasma with or without subsequent silanization. The null hypotheses tested were: (i) argon or oxygen plasma treatments induced no chemical change; (ii) roughness changes on polyethylene fiber surfaces; (iii) exposure time to argon or oxygen plasma treatments had no influence on surface roughness; (iv) silane application induced no chemical change on polyethylene fiber surfaces; (v) argon or oxygen plasma treatments, with or without silanization, had no influence on the flexural strength, and (vi) deflection of a fiber-reinforced composite.

## MATERIAL AND METHODS

**Figure 1:**
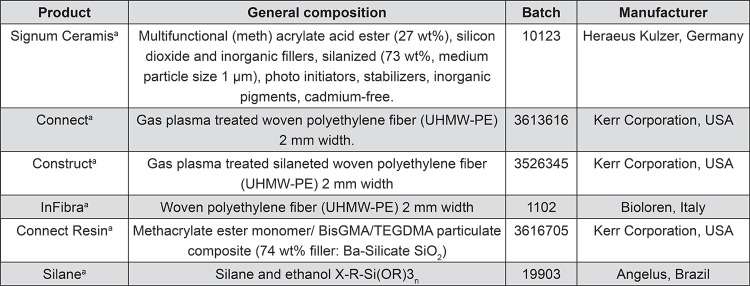
Description of the materials used in the study

^a^ Brand name

Manufacturers and composition of the tested materials are summarized in Figure 1.

BisGMA = 2.2-bis [4-(2-hydroxy-3-methacrylyl-oxyporpoxy) phenyl] propane; TEGDMA = triethyleneglycol-dimethacrylate

### Topographical analysis

Topographical changes were evaluated in six samples (1 cm) of InFibra, Connect, Construct as received, and InFibra treated with oxygen or argon plasma treatment for 1, 3, and 5 min.

The InFibra fibers were subjected to oxygen or argon cold plasma treatment for 1, 3, and 5 min with 30 W power and 1.2x10^-1 ^mbar (12 Pa) pressure in a plasma reactor composed of a borosilicate glass tubular chamber wrapped in a seven turns copper coil. The glass tube has a 3 cm diameter and is 30 cm long; the copper coil has a width of 1 cm. Impedance matching with a 13.56 MHz RF power supply was achieved using a variable capacitor matching box. The initial vacuum was achieved using a mechanical pump connected to the upper part of the reactor. Gas was introduced through a needle valve connected to the lower part of the glass tube. Samples were hung inside the reactor, always in the same position, in such a way that the entire surfaces of the fibers were exposed to the plasma.

All the samples were coated with gold for scanning electron microscopy (SEM) analysis. The JEOL (JSM/6460LV) microscope, with magnifications of 1300x, 3000x, 5000x, 10000x, and 20000x allowed the selection of the best exposition times for the respective gases that induced roughness without inducing major fiber damage.

### Chemical analysis

The effect of cold plasma treatment in the InFibra sample was analyzed by XPS. In total, eight samples (1 cm) of each fiber *per* group were analyzed. The groups analyzed were: Connect, Construct, InFibra, InFibra/Silane, InFibra/O 3 min, InFibra/O 3 min/Silane, InFibra/Ar 3 min, and InFibra/Ar 3 min/Silane.

The chemical characterization of the polyethylene fiber surface was performed using X-ray photoelectron spectroscopy (XPS). The XPS analysis was performed in an ultra-high vacuum station (Escaplus P System, Omicron Nanotechnology, Taunusstein, Hesse, Germany) with a pressure of 10^-10^ mbar in the measurement chamber, using an Al X-ray source (Kα=1486.7 eV), with a 12.5 mA power emission, at a voltage of 16 kV. For the individual peak regions, a pass energy analyzer of 20 eV was used. The survey spectrum was measured at 80 eV pass energy. The binding energies were referred to the carbon 1s level, established as 284.6 eV. Analyses of the peaks were performed with CasaXPS software, using a weighted sum of Lorentzian and Gaussian component curves after Shirley background subtraction. The high resolution spectrum was obtained at 20 eV pass energy, with energy steps of 0.05 eV for the C 1s spectra. All spectra were obtained within one day after treatment.

### Bending tests

Test specimens (N=66) with dimensions of 2×2×25 mm were manufactured and distributed into 11 groups with six samples each. Among the groups, one was an unreinforced control group, and 10 were reinforced with fibers placed at the base of the test specimen (Table 1). Before manufacturing the bending test specimens, 2.5 cm long InFibra fiber strips were subjected to three different treatments: (1) Cold plasma treatment, in which the fibers were exposed to argon or oxygen plasma treatment for 1 or 3 min; (2) Silane application only, in which the fibers were brushed with a thin layer of silane and dried after 1 min with cold air jets for 30 s on each side; and (3) Plasma treatment followed by silane application, in which the fibers were sequentially subjected to the aforementioned processes 1 and 2.

**Table 1:** Report of group, materials, mean flexural strength (σ) in (MPa), mean deflection (D) in (mm) at first load drop and S.D. for groups. Matching superscript letters indicate that there is no statistical difference among the groups (Bonferroni multiple comparison, p<0.05)

Group	Material: Composite, Fibers, and Fiber treatments	Mean flexural strength in MPa (S.D.)	Mean deflection in mm (S.D.)
1	Signum	123.65 (14.79) ^C^	0.027 (0.003) ^D^
2	Signum/Connect	292.19 (44.15) ^A^	0.059 (0.003) ^C^
3	Signum/Construct	237.62 (35.63) ^B^	0.056 (0.014) ^CD^
4	Signum/InFibra	217.67 (14.65) ^B^	0.076 (0.010) ^BC^
5	Signum/InFibra/silane	211.90 (16.57) ^B^	0.094 (0.015) ^AB^
6	Signum/InFibra/O 1 min	265.93 (10.59) ^AB^	0.109 (0.013) ^A^
7	Signum/InFibra/O 3 min	296.27 (24.74) ^A^	0.103 (0.023) ^AB^
8	Signum/InFibra/O 3 min/silane	252.72 (25.48) A^B^	0.089 (0.011) ^AB^
9	Signum/InFibra/Ar 1 min	246.90 (28.49) ^AB^	0.082 (0.011) ^ABC^
10	Signum/InFibra/Ar 3 min	258.82 (21.56) ^AB^	0.076 (0.013) ^BC^
11	Signum/InFibra/Ar 3 min/silane	259.61 (24.26) ^AB^	0.080 (0.029) ^ABC^

All fibers were impregnated with Connect Resin before the sample preparation. The matrix of the unreinforced test specimens was completely filled in one step with the Signum material (control group). In the reinforced test specimens, a single layer of fiber was initially placed, and then, the matrix was filled with Signum material. Photo activation was initially carried out employing a manual photo activator power led Radii-e (SDI - Australia) with 800 mWcm^2^ and wavelength range from 440 to 480 nm in ten 20 s steps, five at the top and five at the base, with the first in the center and the remaining alternating between the right and left sides until reaching the edge of the specimen. Then, the specimen was removed from the matrix and taken to the UniXS (Heraeus Kulzer, Wehrheim, Hesse, Germany) unit for photo activation for 180 s. All test specimens were subjected to manual sanding with 600-grit sandpaper (3M Co.) and stored in distilled water at 37°C for 24 h. The three point bending test was conducted according to ISO 4049:2000(E) in an EMIC DL 2000 material testing machine (test span: 20 mm, crosshead speed of 0.5 mm min^-1^). Load-displacement curves were recorded with PC-computer software (Tesc, v. 1.10, EMIC). Failure was considered to occur at the first load drop higher than 1%, as recommended by the Fracture Mechanics Toughness Test Standard BS 7448 Part 1: 1991 for critical loads in materials presenting pop-ins. From the results of the flexural tests it was possible to calculate the flexural strength and flexural deflection using the following equations:


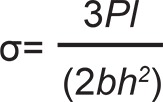


in which σ is the flexural strength, *l* is the span length between the supports, *b * is the sample width, *h* is the sample height, and *P* is the fracture load;


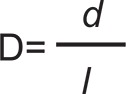


in which *D* is the deflection, *d* is the flexure displacement, and *l* is the span length between the supports.

The mean and standard deviations were calculated. The results were statistically analyzed with ANOVA and the Bonferroni correction for multiple comparison tests at a significance level of p<0.05 using the computer program STATA v. 12.1, 2011.

## RESULTS

### Topographical analysis

**Figure 2:**
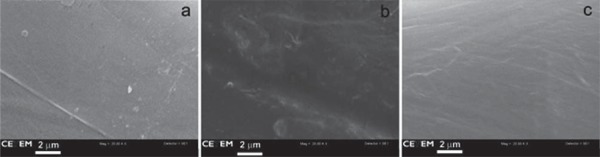
Scanning electron microscopy (SEM) images (x20,000) of (a) Connect, (b) Construct, and (c) InFibra not treated with plasma

**Figure 3:**
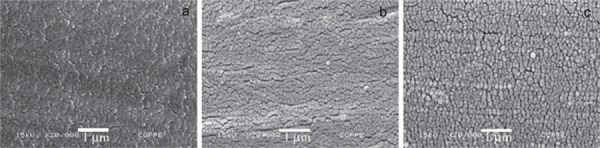
Scanning electron microscopy (SEM) images of InFibra fiber surface treated with argon plasma (x20,000) for: (a) 1 min, (b) 3 min, and (c) 5 min

**Figure 4:**
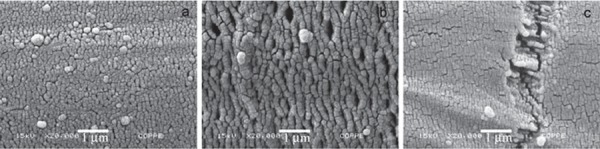
Scanning electron microscopy (SEM) images of InFibra fiber surface treated with oxygen plasma (x20,000) for: (a) 1 min, (b) 3 min, and (c) 5 min

The SEM images with 20000x magnification showed that the surfaces of Connect, Construct, and InFibra fibers were very similar and smooth (Figure 2), and that the treatment of InFibra fiber with argon or oxygen plasma causes surface roughness. Furthermore, it was observed that oxygen plasma is more effective in creating roughness compared to argon plasma (Figures 3 and 4), and that, as the exposure time to plasma increases, the roughness becomes more accentuated.

The use of oxygen plasma for 5 min caused random cracks on the surface, possibly indicating the beginning of damage (Figure 4C). As a result, the chosen treatment times were then 1 and 3 min for both treatments.

### Chemical analysis

Table 2 shows the atomic concentration, obtained through XPS survey spectra, of different chemical elements present in all analyzed groups. It is worth noting that the amount of carbon is less than 80.0% for the Connect and Construct fibers. Both fibers exhibit oxygen bound as C-O or C=O, and Construct fibers also contain silicon. The untreated InFibra sample is mainly composed of carbon (93.4%), with small amounts of oxygen that can be associated to a low level of surface contamination or absorbed species. On the other hand, when the InFibra fibers were treated with oxygen plasma, a great amount of oxygen-based functional groups (26.1%) were incorporated into the fiber surface with the formation of both carbonyl as well as carboxylic groups. Argon plasma induced 14.1% of the incorporation of oxygenated groups to the surface, mainly as C-O.

**Table 2:** Report of XPS analysis: atomic concentration and ratio of peak areas for C 1s of as-received Connect, Construct fibers, and InFibra fiber before and after surface treatment

		Atomic concentration (%)			Relative area under C 1s envelop				
Fiber treatments									
		C 1s	O 1s	Si 2p	C-Si 283.8eV	C-C/C-H 284.8eV	C-O 285.8eV	C=O 287.0eV	O=C-OH 288.5eV
At. (%)	Connect	79.0	21.0	-	-	86.1	8.3	5.6	-
	Construct	78.9	15.1	6.0	-	86.2	10.7	3.1	-
	InFibra	93.4	6.6	-	-	95.3	2.8	1.9	-
	InFibra/Silane	75.6	17.3	7.1	31.8	51.1	10.3	3.6	3.2
	InFibra/O 3 min	73.9	26.1	-	-	34.7	18.5	36.6	10.2
	InFibra/O 3 min/Silane	63.3	29.2	7.5	5.9	49.1	25.6	12.9	6.5
	InFibra/Ar 3 min	85.9	14.1	-	-	77.8	15.5	-	4.7
	InFibra/Ar 3 min/Silane	69.2	22.0	8.8	29.7	39.2	18.8	7.4	4.

Table 2 also shows the chemical groups obtained from the fit of the C 1s XPS high-resolution peak. In surfaces treated with argon plasma, less than 5% of carbonyl groups were observed, and no carboxylic groups could be distinguished by XPS. This may be an indication that argon plasma is less effective than oxygen plasma in inducing superficial oxidation. The incorporation of oxygen into the surface only occurs when the sample is exposed to atmospheric air, and not during the argon plasma processing.

Silane treatment on the InFibra fiber led to the incorporation of 7.1% of silicon atoms. Neither oxygen nor argon plasma induced a great increase in this amount. On the other hand, the incorporation of oxygen increases when samples are pre-treated with oxygen or argon plasma and then, silanized: 17.3% of the oxygen was incorporated into the fiber surface after silane treatment, and this amount increased to 29.2% and 22.0% for fibers pre-treated with oxygen and argon plasma, respectively.

### Bending tests

**Figure 5:**
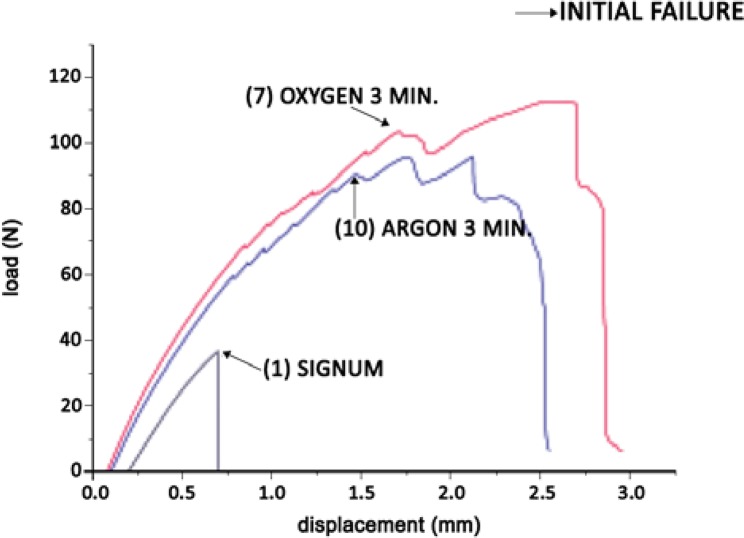
Typical load vs. displacement curves in the flexural test: group (1) unreinforced, and groups (7) and (10) reinforced with fiber. Arrows indicate the first load drop

**Figure 6:**
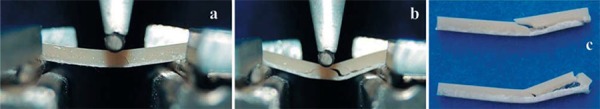
Specimens of fiber reinforced material: (a) close to the first failure point, (b) after major failure, and (c) after the bending tests

During the bending tests, the unreinforced Signum material presented elastic behavior, with only one peak of maximum load to fracture. The test specimens presented brittle fracture in the center. All groups of the reinforced beams presented elasto-plastic behavior with the first maximum load peak followed by displacements associated to load drops and recoveries (Figure 5). All of the test specimens developed one, two, or more cracks in the center of the sample (Figure 6A). Cracks span along the fiber and spread upward, fracturing the Signum material. As the failure process progressed, cracking also occurred close to one of the supports. Then, delamination occurred, separating the Signum material from the fiber on one side of the sample (Figure 6B and C). At the first failure point, where the flexure strength was calculated, all analyzed samples failed only in the central part. None of the samples showed fiber fracture.

The flexural strength and deflection characteristics of the specimens are summarized in terms of their mean values and standard deviations in Table 1.

The flexural strength values of all fiber-reinforced groups were significantly higher than the value of the control group (group 1). The highest strength values were obtained in groups 7 (Signum/InFibra/O 3 min) and 2 (Signum/Connect). These two groups had significant statistical differences when compared to the values of groups 4 (Signum/InFibra), 5 (Signum/InFibra/silane), and 3 (Signum/Construct).

The treatments of the InFibra fiber with argon plasma for 1 and 3 min and oxygen plasma for 1 min promoted an improvement in the behavior of the reinforced material when compared to the material reinforced with untreated InFibra fiber, but it is not statistically significant.

Silane application on the InFibra fiber treated with argon plasma did not change the flexural strength. Silane application on the InFibra fiber not treated or treated with oxygen plasma caused a statistically insignificant reduction in flexural strength.

The deflection of the fiber-reinforced groups, with the exception of the one reinforced with Construct fiber, was significantly larger than the deflection of the control group. The groups reinforced with InFibra fibers presented a large variation of deflection values.

The groups reinforced with InFibra fibers treated with oxygen for 1 and 3 min presented the highest deflection values associated to high flexural strength values. The same did not occur with the groups reinforced with Connect and Construct fibers, in which high flexural strength values were accompanied by low deflection values. The deflection of the samples treated with oxygen for 1 min was significantly larger than those of groups 4 and 10. The application of silane after oxygen plasma treatment led to a decrease in deflection values.

The groups reinforced with InFibra fiber treated with argon plasma for 1 and 3 min, and argon plasma plus silane presented similar mean deflection values, and were very similar to the values of group 4, indicating that treatment with argon plasma, treatment duration, and silane application do not cause significant deflection changes.

## DISCUSSION

As shown in Table 1, the addition of polyethylene fiber results in a significant enhancement of the flexural performance in comparison to the unreinforced dental composite. All of the reinforced groups presented an elasto-plastic behavior, which remained bonded during testing. Catastrophic failure was not observed. In general, these results are in agreement with the results of several other studies^3^ ^,^ ^5^ ^-^ ^7^ ^,^ ^10^ ^,^ ^11^. The fracture of polyethylene fibers was not observed for any group, and this result is in agreement with the work of Karbhari and Strassler^10^ (2007).

The composites reinforced with polyethylene fibers presented a larger deflection, at first load drop, than the unreinforced composite. This was also observed by Karbhari and Strassler^10^ (2007). Karbhari and Wang^11^ (2007) found higher deflection values in the materials reinforced with polyethylene fibers than those found in the present study. These differences may be attributed to the fact that the authors calculated deflection values from the displacement values in the final failure. During the bending tests of the reinforced materials, it was possible to observe the formation of cracks in the composite starting at a certain load. However, the polyethylene fibers held the material together, enabling a relatively ductile behavior and causing a gradual load reduction as the cracks opened until delamination. The delamination was observed to occur between the composite and the fiber with high strain levels. The same behavior was observed by Karbhari and Wang^11^ (2007).

The InFibra fiber treated with oxygen plasma for 3 min significantly improved the mechanical behavior of the fiber-reinforced composite when compared to the composite reinforced with InFibra fiber not treated with plasma, indicating that this treatment promotes a significant improvement of fiber adherence to the Signum material. This result is in agreement with results obtained in other studies^19^ ^,^ ^27^.

Nardin and Ward^20^ (1987) observed that the adherence of the polyethylene fibers treated with oxygen plasma to the epoxy resin is largely increased due to the rough surface structure and chemical bonds between the fiber and the resin. In the present study, utilizing SEM, it was observed that the surface of the untreated InFibra fiber is very smooth, and that the treatments with argon and oxygen created roughness, as observed in other studies^16^ ^,^ ^19^ ^,^ ^28^. As the exposure time to each plasma increased, the roughness became more accentuated^19^ ^,^ ^28^. As observed in the present study by SEM, Connect and Construct fibers, which according to the manufactures are treated with plasma, presented smooth surfaces similar to the surface observed in untreated InFibra fibers, which is unexpected and contradicts our results.

In the present study, SEM images showed that oxygen plasma was more effective than argon plasma, both with a 3 min optimal exposure time, in creating InFibra fiber surface roughness, and improving flexural strength of the fiber-reinforced composite. This indicates that surface roughness is clearly related to the failure mechanism and adherence increase, which is in agreement with other studies^13^ ^,^ ^14^. Unlike the findings of the present study, Tosun, et al.^28^ (2012) verified that argon plasma was more effective than oxygen plasma in promoting surface modifications on the polyethylene fiber. This distinction may be due to the difference in plasma conditions, since low frequency plasma was used in the work of Tosun, et al.^28^ (2012).

The silane application caused a reduction in the strength values of composites reinforced with fibers. This result is in agreement with Ellakwa, et al.^4^ (2002) who observed that the impregnation of Connect fiber with ArtGlass liquid, which contains silane in its formulation, caused a reduction of the strength values of reinforced composites. The results of both studies suggest that the use of silane on polyethylene fibers not treated or treated with oxygen plasma is not recommended. Unlike these findings, Choe and Jang^1^ (1994) verified that epoxy resin composites reinforced with polyethylene fibers treated with plasma and silane presented an increased formation of hydroxyl groups on the surface and higher flexural strength values.

The analysis of the InFibra fiber surface by XPS showed that the treatment with oxygen plasma was more effective than argon plasma in breaking C-C/C-H bonds and incorporating oxygen-containing functional groups into the molecular chain of polyethylene surfaces. Results contrary to these were recently reported by Tosun, et al.^28^ (2012) for polyethylene surfaces after the same plasma treatments. This difference may be attributed to the low frequency plasma employed by Tosun, et al.^28^ (2012). Lee, et al.^16^ (2009) treated polyethylene surfaces with argon or oxygen plasma and reported a similar oxygen group incorporation into the polyethylene molecular chain, as the one reported in the present work. The great amount of oxygen groups (26.1%) incorporated into the fiber surface after oxygen plasma treatment is higher than those presented in the literature^12^ ^,^ ^21^ ^,^ ^22^ ^,^ ^24^ ^,^ ^30^.

The oxygen plasma treatment was efficient in forming chemical functional groups on the fiber surface: C-O (~18%), C=O (~37%), and O-C=OH (~10%). These chemical groups, associated with the micro erosions observed by SEM, promoted an increase in the fiber surface energy and, consequently, an increase in the interface adherence of the fiber to the particulate dental composite. This result was confirmed by a significant increase in flexural strength in group 7. These results are in agreement with Moon and Jang^19^ (1998) and Kusano, et al.^12^ (2011).

The argon plasma treatment also induced the incorporation of oxygen groups, but in a smaller amount: C-O (~15%) and C=O (~5%). The introduction of oxygen is most likely due to the incorporation of O_2_ from room air. Similar results were recently reported for polyethylene surfaces^16^ ^,^ ^28^.

Although XPS results indicated the formation of several silane and hydroxyl functional groups on the surface of the polyethylene fiber in all groups treated with silane, this treatment did not improve the flexural strength values of composites reinforced with fibers in any case. These observations could be an indication that silane application on polyethylene fibers is not effective.

## CONCLUSIONS

Based on the results of this study, all the null hypotheses were rejected and it can be concluded that:

(1) Both oxygen and argon cold plasma treatments induce roughness on the polyethylene fiber surface structures. As the exposure time for the plasma treatment increases, for both oxygen and argon, roughness is accentuated. Oxygen plasma is more effective.

(2) Oxygen and argon cold plasma treatments were effective in incorporating hydroxyl as well as oxygenated functional groups. This incorporation was more significant for oxygen plasma, in which the oxygen amount was similar to the amount observed for the Connect fiber.

(3) The application of silane on polyethylene fibers treated with argon or oxygen cold plasma or untreated fibers, despite introducing chemical functional groups on the surface, does not promote an increase in flexural strength of the reinforced composites.

(4) The argon or oxygen cold plasma treatment affected the flexural strength and deflection of a fiber-reinforced composite. The highest strength values were obtained in the group reinforced with polyethylene fibers treated with oxygen plasma for 3 min. The groups reinforced with polyethylene fibers treated with oxygen for 1 and 3 min presented the highest deflection values.

(5) The increase in roughness, as well as the incorporation of oxygenated functional groups, may be the key factor for the significant increase in the mechanical properties of composites reinforced with polyethylene fibers treated with oxygen cold plasma for 3 min.

## References

[1] Choe CR, Jang J (1994). Surface modification of HS/HM polyethylene fiber for composite applications (I). Korea Polymer J.

[2] Deshmukh RR, Shetty AR (2007). Modification of polyethylene surface using plasma polymerization of silane. J Appl Polym Sci.

[3] Dyer RS, Lassila LVJ, Jokinen M, Vallittu PK (2004). Effect of ﬁber position and orientation on fracture load of ﬁber-reinforced composite. Dent Mater.

[4] Ellakwa AE, Shortall AC, Marquis PM (2002). Inﬂuence of ﬁber type and wetting agent on the ﬂexural properties of an indirect ﬁber reinforced composite. J Prosthet Dent.

[5] Ellakwa AE, Shorthall AC, Marquis PM (2003). Influence of fibre position on the flexural properties and strain energy of a fibre-reinforced composite. J Oral Rehabil.

[6] Ellakwa AE, Shorthall AC, Shehata MK, Marquis PM (2001). The influence of fibre placement and position on the efficiency of reinforcement of fibre reinforced composite bridgework. J Oral Rehabil.

[7] Ellakwa AE, Shorthall AC, Shehata MK, Marquis PM (2001). Influence of veneering composite composition on the efficacy of fiber-reinforced restorations (FRR). Oper Dent.

[8] Ellakwa AE, Shorthall AC, Shehata MK, Marquis PM (2002). Influence of bonding agent composition on flexural properties of an Ultra-High Molecular Weight Polyethylene Fiber-Reinforced Composite. Oper Dent.

[9] Freilich MA, Meiers JC, Duncan JP, Goldberg AJ (2000). Fiber-reinforced composites.

[10] Karbhari VM, Strassler H (2007). Effect of fiber architecture on flexural characteristics and fracture of fiber-reinforced dental composites. Dent Mater.

[11] Karbhari VM, Wang Q (2007). Inﬂuence of triaxial braid denier on ribbon-based ﬁber reinforced dental composites. Dent Mater.

[12] Kusano Y, Teodoru S, Hansen CM (2011). The physical and chemical properties of plasma treated ultra-high-molecular-weight polyethylene fibers. Surf Coat Technol.

[13] Ladizesky NH (1990). The integration of dental resins with highly drawn polyethylene fibres. Clin Mater.

[14] Ladizesky NH, Ward IM (1989). The adhesion behavior of high modulus polyethylene fibres following plasma and chemical treatment. J Mater Sci.

[15] Ladizesky NH, Ward IM (1995). A review of plasma treatment and the clinical application of polyethylene fibres to reinforcement of acrylic resins. J Mater Sci Mater Med.

[16] Lee JH, Rhee KY, Lee JH (2009). Effects of reactive gas on shear and fracture behaviors of plasma-treated polyethylene/steel joints. Appl Surf Sci.

[17] Liston EM, Martinu L, Wertheimer MR (1993). Plasma surface modification of polymers for improved adhesion: a critical review. J Adhes Sci Technol.

[18] Miller A, Schwartz P (1997). Effects of oven aging on plasma treated ultra high strength polyethylene. Plasmas Polym.

[19] Moon SI, Jang J (1998). Factors affecting the interfacial adhesion of ultrahigh-modulus polyethylene fibre vinylester composites using gas plasma treatment. J Mater Sci.

[20] Nardin M, Ward IM (1987). Influence of surface-treatment on adhesion of polyethylene fibres. Mat Sci Technology.

[21] Ogawa T, Mukai H, Osawa S (1999). Effects of functional groups and surface roughness on interfacial shear strength in ultrahigh molecular weight polyethylene fiber/polyethylene system. J Appl Polym Sci.

[22] Qiu Y, Zhang C, Hwang Y J, Bures BL, McCord M (2002). The effect of atmospheric pressure helium plasma treatment on the surface and mechanical properties of ultrahigh-modulus polyethylene fibers. J Adhes Sci Tech.

[23] Rashidan N, Esmaeili V, Alikhasi M, Yasini S (2010). Model system for measuring the effects of position and curvature of fiber reinforcement within a dental composite. J Prosthodont.

[24] Ren Y, Wang C, Qiu Y (2008). Aging of surface properties of ultra-high modulus polyethylene fibers treated with He/O2 atmospheric pressure plasma jet. Surf Coat Technol.

[25] Sirisinha K, Chimdist S (2006). Comparison of techniques for determining crosslinking in silane-water crosslinked materials. Polym Test.

[26] Spyrides SM, Bastian FL (2004). In vitro comparative study of the mechanical behavior of a composite matrix reinforced by two types of fibers (polyethylene and glass). Mater Sci Eng C.

[27] Tissington B, Pollard G, Ward IM (1991). A study of the influence of fibre/resin adhesion on the mechanical behavior of ultra-high-modulus polyethylene fibre composites. J Mater Sci.

[28] Tosun K, Felekoğlu B, Baradan B (2012). Multiple cracking response of plasma treated polyethylene fiber reinforced cementitious composites under flexural loading. Cem Concr Compos.

[29] Wang J, Smith KJ (1999). The breaking strength of ultra-high molecular weight polyethylene fibers. Polymer.

[30] Wang T, Wang C, Qiu Y (2008). Surface modification of ultra high modulus polyethylene fibers by an atmospheric pressure plasma jet. J Appl Polym Sci.

